# Acute Tubulointerstitial Nephritis (ATIN) in Patient With Autosomal Dominant Polycystic Kidney Disease (ADPKD): A Case Report

**DOI:** 10.1155/crin/3069446

**Published:** 2025-08-23

**Authors:** Mohammad Alsultan, Marwa Kliea, Alaa Aldin Zedan, Qussai Hassan

**Affiliations:** ^1^Department of Nephrology, Faculty of Medicine, Damascus University, Damascus, Syria; ^2^Department of Neurology, Faculty of Medicine, Damascus University, Damascus, Syria; ^3^Department of Internal Medicine, Burjeel Hospital for Advanced Surgery, Dubai, UAE

**Keywords:** acute kidney injury (AKI), acute tubulointerstitial nephritis (ATIN), autosomal dominant polycystic kidney disease (ADPKD), ciprofloxacin, corticosteroids (CS)

## Abstract

**Background:** Autosomal dominant polycystic kidney disease (ADPKD) is characterized by diffuse renal cysts that secrete cytokines, which induce interstitial inflammation and fibrosis. Meanwhile, acute tubulointerstitial nephritis (ATIN) is characterized by inflammatory infiltrates in the interstitium, where kidney biopsy remains the mainstay for diagnosis.

**Case Presentation:** An 85-year-old male complained of fatigue, loss of appetite, and low-grade fever for a week. Within the past month, the patient received ciprofloxacin for a urinary tract infection (UTI) and described flu symptoms. The medical history consisted of ADPKD type 2 with baseline serum creatinine (sCr) at 1.2 mg/dL. Labs showed acute kidney injury (AKI) (sCr = 3.98 mg/dL). The combination of previous drug and infection exposure, systemic symptoms, and AKI suggested the diagnosis of ATIN. The kidney function and clinical status improved with corticosteroids (CS) treatment, where sCr returned to 2.4 mg/dL. Unfortunately, the patient died due to severe community-acquired pneumonia.

**Conclusion:** This case highlighted the dilemma of ATIN diagnosis in a patient with ADPKD and presents the first case of ATIN in ADPKD patients. Kidney biopsy was unable to be performed for ATIN diagnosis in this ADPKD patient due to diffuse renal cysts. Also, the biopsy could be confused by interstitial fibrosis and infiltrates that appeared early in ADPKD biopsies. Clinicians could suggest an ATIN diagnosis and start treatment based on the combination of new-onset AKI aligned with clinical history and laboratory tests in such ADPKD patients. Also, the improvement of kidney function after CS treatment could support the ATIN diagnosis.

## 1. Introduction

Autosomal dominant polycystic kidney disease (ADPKD) is a multisystem disorder characterized by multiple, bilateral renal cysts and associated with cysts in other organs, with a prevalence estimated at 1 in 400 to 1 in 1000 [1]. There are two principal causative genes that have been identified, *polycystin 1* (PKD 1) and *polycystin 2* (PKD 2), with strikingly high phenotypic and intrafamilial variability, which causes a wide range in age at presentation, severity of disease, and time to reach end-stage renal disease (ESRD) [1, 2]. Usually, mutations in PKD 2 lead to much milder disease compared to PKD 1 with average ages at ESRD of 79.7 and 58.1 years, respectively [2]. The diagnosis of ADPKD relies mainly on imaging, which reveals large kidneys with multiple bilateral cysts [1, 2]. The pathology of ADPKD showed an increasing number and size of cysts that arise from all segments of the nephron and collecting ducts [1]. The cystic epithelium secretes large amounts of cytokines, which induce inflammation and fibrosis surrounding the cysts [2]. Also, there is an advanced sclerosis of preglomerular vessels, interstitial fibrosis, and tubular epithelial hyperplasia even at the early stages of the disease [1]. Other extrarenal manifestations of ADPKD have been described including hypertension (HTN), polycystic liver disease, intracranial aneurysms, and valvular heart disease [1].

Acute tubulointerstitial nephritis (ATIN) is an acute hypersensitivity reaction type prescribed intravenous (IV) characterized by inflammatory infiltrates in the kidney interstitium [1]. ATIN represents a relatively uncommon cause of acute kidney injury (AKI), with an incidence accounting for 2% to 3% of all renal biopsies; however, the incidence may increase [1, 3]. The etiologies associated with ATIN include drugs, infections, autoimmune, and idiopathic. Drug-induced ATIN (DI-ATIN) accounts for about 70% to 90% of all cases [1, 3]. ATIN diagnosis is usually challenging because most patients are asymptomatic or present with nonspecific symptoms including fever, nausea, vomiting, diarrhea, abdominal pain, myalgias, and rash [3]. Clinical history can often give clues to the possibility and etiology of ATIN [1]. Since there is a latent period between exposure and presentation, patients can miss the new exposure to inciting agents or infections [1]. Thus, ATIN diagnosis should be considered in any patient with unexplained AKI [1, 3]. When there are no definitive laboratory tests to diagnose ATIN, kidney biopsy remains the utility of choice for ATIN diagnosis [1, 3].

Here, we reported a challenging case of ATIN in a patient with ADPKD. When the kidney biopsy was unable to be performed due to diffuse cortical cysts, the diagnosis was made clinically and supported by improvement after corticosteroids (CS) treatment.

## 2. Case Presentation

An 85-year-old male complained of fatigue, loss of appetite, and low-grade fever for a week. The patient received ciprofloxacin (for 10 days) for a urinary tract infection (UTI) in the past month and described flu symptoms (nasal congestion and myalgias) in the past 2 weeks.

The medical history consisted of ADPKD type 2 (diagnosed at the age of 60 years), hypertension, coronary artery bypass grafting, and cerebellar infarction. His son was also diagnosed with ADPKD and HTN at fourth decades; however, there are no genetic tests to know the affected genes. The patient received valsartan, amlodipine, atorvastatin, bisoprolol, clopidogrel, apixaban, trimetazidine, and vitamins. The basal serum creatinine (sCr) was 1.2 mg/dL (in the past month), which equals a glomerular filtration rate (GFR) of 59 mL/min/1.73 m^2^.

**Figure 1:**
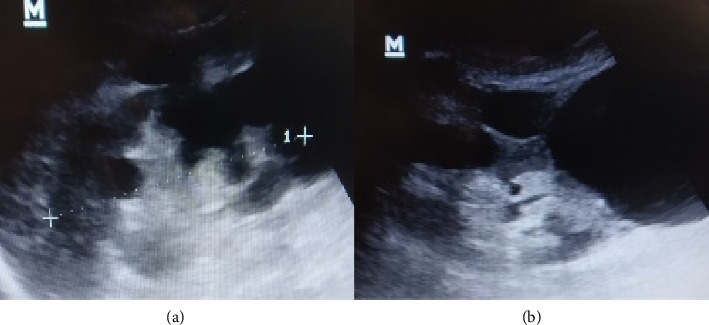
Kidneys ultrasound; showed multiple and diffuse cysts in both left (a) and right (b) kidneys.

Labs showed AKI, where sCr = 3.98 mg/dL (Table 1). Kidney ultrasound (US) showed diffuse cysts consistent with ADPKD (Figure 1). There was no dehydration (prerenal azotemia), no prostatic hypertrophy or urinary occlusion (postrenal azotemia), and no previous exposure to nephrotoxic drugs (such as aminoglycosides or contrast agents).

The combination of previous drug and infection exposure, systemic symptoms, and AKI suggested the diagnosis of ATIN. Also, the ophthalmic examination did not show uveitis. The kidney biopsy could not be performed due to ADPKD. When the drug is suspected to cause ATIN, the main treatment consists of prompt discontinuation of the offending agent; however, our patient received the drug (ciprofloxacin) in the past month. Also, we monitored the kidney function for 2 weeks after the first examination and excluded other reasons like immune disorders (Table 1).

When kidney function did not return to baseline after drug discontinuation and monitoring the kidney function for 2 weeks, most nephrologists prescribed a corticosteroid (CS) course [3, 4]. Also, several centers do not wait and start a short course of CS for suspected ATIN, even without biopsy confirmation [3, 4].

So, we IV methylprednisolone 0.5 g for 3 days, followed by oral prednisone (1 mg/kg/days). The patient was discharged after a week with outpatient monitoring every week for kidney function tests, blood glucose, urinary output, and clinical status. sCr at discharge was 3.5 mg/dL and improved to 2.9 mg/dL in the second week. The clinical status improved, where fatigue, appetite, and fever were resolved and urinary output was increased. The kidney function and clinical status improved, and the sCr returned to 2.4 mg/dL by 3 weeks of treatment. Unfortunately, the patient died in the following week (the fourth week of treatment) due to severe community-acquired pneumonia.

## 3. Discussion

Given the recent history of antibiotic use followed by a rise in creatinine and a decrease in creatinine following steroid treatment, it is reasonable to believe this is a case of drug-induced acute TIN complicating ADPKD, which has not been reported previously. ADPKD patients demonstrate prominent interstitial fibrosis aligned with interstitial infiltrates of macrophages and lymphocytes even with normal renal function or early renal failure [1].

This case highlighted the dilemma of ATIN diagnosis in a patient with ADPKD. First, kidney biopsy is avoided in ADPKD patients due to the presence of diffuse cortical cysts. Second, when interstitial fibrosis and infiltrates can appear early in ADPKD biopsies, kidney biopsy cannot approve ATIN diagnosis in ADPKD patients unless a new history of AKI appears and other AKI etiologies are excluded.

Most patients with ATIN could present with an incidental and subacute rise in sCr with minimal symptoms or no symptoms at all, which could delay the diagnosis and lead to fibrosis and the development of chronic kidney injury [3, 5]. This is mainly observed in DI-ATIN, specifically NSAIDs and PPIs, which might cause ATIN after several months of treatment initiation [1, 5]. Also, fluoroquinolones, especially ciprofloxacin, are among the most common causative agents of ATIN, which is characterized by delayed onset of clinical manifestations, and making the diagnosis is a challenging task [3].

On the other hand, many infectious pathogens can cause renal parenchymal inflammation by direct infection, such as in acute pyelonephritis, whereas other infections may induce an immunologically mediated ATIN in the absence of direct invasion [1, 3]. Viruses could cause kidney injury as a consequence of direct viral infection (toxic tubulopathy) or secondary to viral-induced immune response [3].

ATIN presents with elevated sCr and abnormal urinalysis (pyuria, hematuria, and eosinophiluria) aligned with elevated ESR, anemia, and eosinophilia [3, 5]. None of these tests are specific for ATIN diagnosis, and renal biopsy remains the most accurate method to diagnose [1, 3, 5]. However, in cases where the kidney biopsy is unable to be performed, clinical history aligned with abnormal serum and urine tests could often give clues to the possibility and etiology of ATIN diagnosis [1, 3].

Here, the patient described exposure to ciprofloxacin for 10 days for UTI treatment in the past month and symptoms of upper respiratory tract infection (flu symptoms) in the past 10 days. So, the etiology of ATIN could be ciprofloxacin or viral infection. When we could not perform the kidney biopsy due to diffuse cortical cysts, the diagnosis was made clinically by the new onset of AKI with abnormal urinalysis and excluding immunologic disorders. Thereafter, CS improved the clinical status and kidney function, which further supported the diagnosis of ATIN. This practice in such ADPKD patients seemed reasonable in light of kidney biopsy inability. Similarly, a study by Taktak et al. [4] reported ATIN diagnosis in 19 pediatric patients, where kidney biopsy was performed in only five patients, and 14 patients were diagnosed clinically by a combination of the history, symptoms, and laboratory findings.

The cornerstone of ATIN treatment is mainly identification and prompts discontinuation of the inciting agent [5]. Most experts initially give a high dose of CS, either intravenous or oral, followed by gradual tapering for a total of 4 to 6 weeks, and some prolong the treatment for 3 to 6 months as the patient responds [3, 5]. Historically, ATIN has a good prognosis with complete recovery of renal function; however, recent studies show that sCr does not return to baseline in about 40% to 50% of cases [6, 7]. Even in those with renal recovery, an increase in sCr can persist for several weeks [1].

This patient showed a gradual kidney function improvement with a slow reduction of sCr after 3 weeks of CS treatment; this might be due to the underlying chronic injury belonging to ADPKD and delaying the ATIN diagnosis.

## 4. Conclusion

Clinicians could suggest ATIN diagnosis and start treatment by the combination of new-onset AKI aligned with clinical history of inciting agent, symptoms, and laboratory tests in such ADPKD patients. Also, the improvement of kidney function after CS treatment could support the ATIN diagnosis.

## Figures and Tables

**Table 1:** Laboratory tests at presentation.

WBC (× 10^3^/mm^3^)	Hb (g/dL)	CRP (mg/dL)	Ur (mg/dL)	Cr (mg/dL)	K (mEq/L)	ESR mm/h	C3 (mg/dL)
7.4	9.6	0.5	173	3.98	5.5	40	115

**C4 (mg/dL)**	**Ferritin (ng/mL)**	**Trans-Sat (%)**	**Urine**	**WBC cells/HPF**	**RBC cells/HPF**	**Prot**	**Culture**

32	19	12%		16	10	+	Neg

## Data Availability

The data are available from the corresponding author upon reasonable request.

## References

[1] Johnson R. J. (2024). *Comprehensive Clinical Nephrology*.

[2] Chebib F. T., Torres V. E. (2016). Autosomal Dominant Polycystic Kidney Disease: Core Curriculum 2016. *American Journal of Kidney Diseases*.

[3] Atta M. G. (2022). *Tubulointerstitial Nephritis*.

[4] Taktak A., Uncu N., Acar B. (2015). Acute Tubulointerstitial Nephritis: A Case Series and Long-Term Renal Outcomes. *Turkish Journal of Pediatrics*.

[5] Lerma E. V. (2018). *Current Diagnosis & Treatment. Nephrology & Hypertension*.

[6] Muriithi A. K., Nasr S. H., Leung N. (2013). Utility of Urine Eosinophils in the Diagnosis of Acute Interstitial Nephritis. *Clinical Journal of the American Society of Nephrology*.

[7] Muriithi A. K., Leung N., Valeri A. M. (2014). Biopsy-Proven Acute Interstitial Nephritis, 1993–2011: A Case Series. *American Journal of Kidney Diseases*.

